# A Curry-style Semantics of Interaction: From Untyped to Second-Order Lazy $\lambda \mu$-Calculus

**DOI:** 10.1007/978-3-030-45231-5_22

**Published:** 2020-04-17

**Authors:** James Laird

**Affiliations:** 8 ENS/CNRS, Université Paris-Saclay, Cachan, France; 9 University of Duisburg-Essen, Duisburg, Germany; Department of Computer Science, University of Bath, Bath, UK

## Abstract

We propose a “Curry-style” semantics of programs in which a nominal labelled transition system of types, characterizing observable behaviour, is overlaid on a nominal LTS of untyped computation. This leads to a notion of program equivalence as typed bisimulation.

Our semantics reflects the role of types as hiding operators, firstly via an axiomatic characterization of “parallel composition with hiding” which yields a general technique for establishing congruence results for typed bisimulation, and secondly via an example which captures the hiding of implementations in abstract data types: a typed bisimulation for the (Curry-style) lazy $\lambda \mu$-calculus with polymorphic types. This is built on an abstract machine for CPS evaluation of $\lambda \mu$-terms: we first give a basic typing system for this LTS which characterizes acyclicity of the environment and local control flow, and then refine this to a polymorphic typing system which uses equational constraints on instantiated type variables, inferred from observable interaction, to capture behaviour at polymorphic and abstract types.

## 1 Introduction

"Church-style" and "Curry-style" are used to distinguish programming languages in which the type of a term is intrinsic to its definition from those in which it is an extrinsic property. The same distinction may be applied to semantics of programming languages: in many models, type-objects are essential to the interpretation of a term — e.g. as a morphism between objects (types) in a category — but interpreting terms independently of their types (as in e.g. realizability interpretations) may have conceptual and practical advantages, particularly for describing Curry-style type systems. The aim of this semantic investigation of higher-order programs is to develop a Curry-style semantics of interaction by overlaying a labelled transition system of types onto a LTS of untyped computation, so that the observable behaviour of a typed state is restricted to the actions made available by its type. Our objective is to apply this to lazy functional programs: untyped and with Curry-style polymorphic typing systems, and to develop a theory of program equivalence — *typed bisimulation* — able to describe genericity and abstract datatypes in this setting.

**Game Semantics** Games models for programming languages are typically (but not invariably) given in a Church-style: terms are interpreted as strategies on a specified two-player game which represents their type [2,9]. This kind of semantics is compositional by definition, at the cost of forgetting the internal computational behaviour of programs, and potentially excluding system level behaviour [6]. It uses categorical structure to describe its models and prove key results — in particular *soundness* with respect to an operational semantics.

By contrast, in *operational* game semantics [15,12], programs are interpreted as states in a labelled transition system based directly on their syntax and operational semantics. Internal computation is retained but can be factored out by restricting to observable behaviour. Soundness of these models "comes for free" — instead, the fundamental property requiring non-trivial proof is that they are *compositional* — that is, the equivalence induced on programs is a congruence. Basic structure which supports and systematizes these proofs would be useful (techniques such as Howe's method are not available in this intensional setting). We aim to show that defining operational game semantics in a Curry style gives the opportunity to formulate and apply such structure. This is complementary to characterization of the structure of operational game semantics at a categorical level [18], into which we believe our semantics can fit well. Our motivation and general methodology bears similarities to the programme of Berger, Honda and Yoshida [3] —- in which Curry-style types are used to characterize the $\pi$-calculus processes corresponding to functional and polymorphic programs — and to typing systems for process calculi such as those described in [10].

**Hiding using types** We will interpret (extrinsic) types as hiding operators: windows through which terms of a given type may interact with the world, while their internal behaviour is hidden from external observation — both passive and active. Our goal is to show that this interpretation can be used to model information hiding in two key areas of higher-order computation. The first, "parallel composition with hiding" is the fundamental operation on which game semantics is based. We axiomatize the notion of a typing system for an LTS with such an operation, in which a type is a state which characterizes precisely the possible interaction between a function and its argument at that type.

The second form of information hiding for which we give a Curry-style interpretation is hiding of implementation details using polymorphic (existential) types as abstract data types. Our key example of a typed labelled transition systems is a new model of the second-order $\lambda\mu$-calculus: we shall now discuss the background and significance of this contribution.

## 1.1 Program Equivalence and Polymorphism

Our starting point is the lazy $\lambda$-calculus — the pure, untyped $\lambda$-calculus, evaluated by weak head reduction — and its extension with first-class continuations, the corresponding version of Parigot's $\lambda\mu$-calculus [21]. As argued in [1], the lazy $\lambda$-calculus approximates well to the behaviour of lazy functional programming languages such as Haskell, and is thus an appropriate setting in which to explore properties such as program equivalence, for which there is now a rich and well-studied theory. For instance, *open* or *normal form* bisimilarity [25] is a coinductively defined equivalence which extends $\beta$-equivalence to infinitary behaviours. It gives a purely intensional characterization of program equivalence (by contrast to e.g. applicative bisimilarity, which involves quantifying over all possible arguments) and has a variety of alternative characterizations — for instance two terms are open bisimilar if and only if they have the same *Levy-Longo* trees [19], or their (call-by-name) translations in the $\pi$-calculus are weakly bisimilar [25,5]. (Or, indeed, if they are normal-form bisimilar as $\lambda\mu$-terms.)

Normal form bisimilarity of simply-typed $\lambda$-terms is just $\beta$-equivalence. However, extending to polymorphic types, such as those of the second-order $\lambda$-calculus (System F) [7,24] poses deeper questions. A primary motivation for introducing polymorphic types is that they can express abstract data types which hide implementation details [20] (cf. the module systems of Haskell and ML). A useful notion of program equivalence should therefore reflect this. As a simple example, the untyped $\lambda$-terms $\lambda f.f\,\lambda x.\lambda y.x$ and $\lambda f.f\,\lambda x.\lambda y.y$ are clearly not normal form bisimilar. But at the second-order type $\exists X.X \triangleq \forall Y.(\forall X.X \to Y) \to Y$ (which they both inhabit in a Curry-style presentation), they should be behaviourally equivalent — since any function of type : $\forall X.(X \to Y)$ will never call its argument. In other words, the existential type $\exists X.X$ "hides" the difference between $\lambda f.f\,\lambda x.\lambda y.x$ and $\lambda f.f\,\lambda x.\lambda y.y$. This is an observational equivalence, but of a particularly fundamental kind, since it (and other equivalences involving abstract data types) is robust in the presence or absence of side-effects. It can be captured by extensional methods such as applicative bisimilarity, which was extended to a polymorphic setting in [26], but this requires *quantification* over instantiating terms and types, whereas our semantics is based on *unification* of instantiating types.

The problem is that comparing the evaluation trees of terms (e.g. by normal form bisimulation) does not capture the capacity of their types to restrict interaction with the environment. Game semantics does reflect this interaction (in various manifestations), and therefore offers a potential solution. Although several games models for polymorphism do not capture data abstraction by existential types (including Hughes' semantics of System F [8], which is faithful with respect to $\beta\eta$-equivalence, and Curry-style models [16]) a series of related approaches does so. These include translation into the (polymorphically typed) $\pi$-calculus [4], and an operational form [17,27] and a traditional compositional presentation [14,13] of game semantics.

In these semantics, values of polymorphic variable type are interpreted as *pointers* to data of undisclosed type — e.g. a location where it is stored, or a channel on which it may be received. Instantiation of universally quantified type variables replaces this pointer-passing with copycat behaviour. This gives a natural interpretation of polymorphism in settings such as the $\pi$-calculus, or languages with general references, where pointers are first-class objects. However, it is closely associated with a Church-style presentation of second-order type systems — e.g. by the interpretation of type abstraction as an explicit creation of a pointer; in the case of "typed normal form bisimulation" [17] the translation of a term is explicitly determined by its type. This is significant because it is in the presence of polymorphism that key differences between Church-style and Curry-style emerge — for example, in allowing intersection types. The pointer-passing models also exhibit behaviours which go beyond untyped functional interaction, making their relationship to it unclear — in the game semantics [14], instantiation violates the fundamental innocence and visibility conditions on strategies; the $\pi$-calculus interpretation uses free name as well as bound name passing.

Curry-style semantics give a natural interpretation of second-order Curry-style typing, with a simple relationship to the semantics of the untyped $\lambda\mu$-calculus, by overlaying a more refined LTS of second order types on the same underlying LTS of computations.

## 2 Typed Labelled Transition Systems

In this section we describe a notion of typed labelled transition system and an associated equivalence: typed bisimulation. Based on this we axiomatize a simple typing system for parallel composition with hiding and show that it preserves typed bisimulation. Examples of typed LTS (in the form of models of the lazy $\lambda\mu$-calculus and lazy $\lambda\mu 2$-calculus) follow in the rest of the paper.

We work in the setting of *nominal sets* [23], which allows the introduction of fresh names (for store locations, communication channels, types etc). Assume a fixed, infinite set of *atoms* and a group $G$ of permutations on them. A nominal set $X$ is an action of $G$ on a set $|X|$ such that each $x \in |X|$ has a finite supporting set of atoms such that if $\pi(a) = a$ for all atoms in this set then $\pi \cdot x = x$. We write $\sup(x)$ for the $\subseteq$-least of these sets (which is the intersection of all supporting sets for $x$).

**Definition 1.** *A nominal LTS is a labelled transition system $(\mathcal{S}, Act, \rightarrow)$ such that $\mathcal{S}$ (states) and Act (actions) are nominal sets and the transition relation $\rightarrow$ is* equivariant *— i.e. for any $\pi \in G$, $C \xrightarrow{a} C'$ if and only if $\pi \cdot C \xrightarrow{\pi \cdot a} \pi \cdot C'$.*

Similarly motivated notions of nominal LTS are developed in e.g. [22]. Our key example — an abstract machine for direct-style CPS evaluation — is given in the next section.

The directly observable part of a labelled transition system may be characterized by defining a *typing system* for it. (Similar notions of typing system for a process calculus are defined in [10], for example.)

**Definition 2.** *A* typing system *for a nominal LTS $(\mathcal{S}; Act; \rightarrow)$ is a nominal LTS $(\mathcal{T}; Obs; \hookrightarrow)$ such that $Obs \subseteq Act$, with a relation, $⦂$ (typing), from $\mathcal{S}$ to $\mathcal{T}$ which satisfies the following* subject reduction *properties for each $C ⦂ T$:*

- *If $C \xrightarrow{a} C'$ and $T \overset{a}{\hookrightarrow} T'$ then $C' ⦂ T'$ (we write $C ⦂ T \xrightarrow{a} C' ⦂ T'$).*
- *If $C \xrightarrow{a} C'$, where $a \notin Obs$ and* $\sup(C') \cap \sup(T) \subseteq \sup(C) \cap \sup(T)$, *then $C' ⦂ T$ (we write $C ⦂ T \longrightarrow C' ⦂ T$).*

Subject reduction requires that actions which are *observable* (i.e. in $Obs$) change a computation and its type in a way that respects the typing relation, and that those which are *internal* to a computation (i.e. in $Act \backslash Obs$) maintain its type (provided that any names fresh for the state are also fresh for its type).

Let $\Longrightarrow$ be the reflexive, transitive closure of the internal reduction $\longrightarrow$, and define $C \mathbin{⨾} T \stackrel{a}{\Longrightarrow} C' \mathbin{⨾} T'$ if $C \mathbin{⨾} T \Longrightarrow D \mathbin{⨾} T \stackrel{a}{\longrightarrow} D' \mathbin{⨾} T' \Longrightarrow C' \mathbin{⨾} T'$. To define weak bisimulation between typed states based on these relations, we need to take account of the fact that a name may be fresh for one, but already occur internally in the other (cf. [22]). So bisimulation is defined up to the equivalence on the states of type $T$ which allows permutation of internal names: $C \simeq_T C'$ if there exists a permutation $\pi \in \mathrm{stab}(T)$ (i.e. $\pi \cdot T = T$) such that $C' = \pi \cdot C$.

**Definition 3.** *A* typed bisimulation *is a binary, symmetric, equivariant relation* $\mathcal{R}$ *between typed states* $(C \mathbin{⨾} S)$, *such that if* $(C \mathbin{⨾} S)\mathcal{R}(D \mathbin{⨾} T)$ *then* $S = T$ *and:*

1. *If* $C \mathbin{⨾} T \stackrel{a}{\longrightarrow} C' \mathbin{⨾} T'$ *then there exists* $D' \simeq_T D$ *such that* $(D' : T) \stackrel{a}{\Longrightarrow} (D'' : T')$, *where* $(C' \mathbin{⨾} T')\mathcal{R}(D'' : T')$.
2. *If* $C \mathbin{⨾} T \longrightarrow C' \mathbin{⨾} T$ *then there exists* $D' \simeq_T D$ *such that* $(D' : T) \Longrightarrow (D'' : T)$, *where* $(C' \mathbin{⨾} T)\mathcal{R}(D'' : T)$.

*Typed bisimilarity is the largest typed bisimulation: states* $C$ *and* $D$ *are bisimilar at type* $T$ $(C \sim_T D)$ *if* $(C \mathbin{⨾} T)$ *and* $(D \mathbin{⨾} T)$ *are typed bisimilar.*

### 2.1 Parallel Composition with Hiding

Having proposed an interpretation of types as operators which hide internal communication, we now characterize the properties of a typing system for *parallel composition with hiding* which entail that it preserves typed bisimulation (i.e. the latter is a congruence).

**Definition 4.** *An* interaction structure *is a nominal LTS* $(\mathcal{S}; Act; \rightarrow)$ *such that* $Act = \mathcal{L} \cup (\{+,-\} \times \mathcal{L})$ *for some set of* $\mathcal{L}$ *of (unpolarized) labels, with an equivariant partial binary operation* $|$ *on* $\mathcal{S}$ *(*parallel composition*) such that if* $C = C_1|C_2$ *then* $C \stackrel{a}{\longrightarrow} C'$ *if and only if* $C' = C_1'|C_2'$ *for some* $C_1'$ *and* $C_2'$ *such that either:*

- $C_1 \stackrel{a}{\longrightarrow} C_1'$ *and* $C_2' = C_2$, *where* $(\mathrm{sup}(C_1') \cup \mathrm{sup}(a)) \cap \mathrm{sup}(C_2) \subseteq \mathrm{sup}(C_1)$ *or,*
- $C_1' = C_1$ *and* $C_2 \stackrel{a}{\longrightarrow} C_2'$, *where* $(\mathrm{sup}(C_2') \cup \mathrm{sup}(a)) \cap \mathrm{sup}(C_1) \subseteq \mathrm{sup}(C_2)$ *or,*
- $C_1 \stackrel{pa}{\longrightarrow} C_1'$ *and* $C_2 \stackrel{\overline{p}a}{\longrightarrow} C_2'$, *where* $p \in \{+,-\}$.

The nominal side-conditions require that any names which are fresh for the component to which they are introduced are fresh for the whole state.

Parallel composition is typed using a *ternary relation* between types: $T_1 \stackrel{T_2}{\multimap} T_3$ means "$T_2$ is an arrow type from $T_1$ to $T_3$" — there may be several arrow types between two types (or none).

**Definition 5.** *A typing system for an interaction structure* $(\mathsf{Comp}, \mathcal{L}, |)$ *is a typing system* $(\mathcal{T}; (\{+,-\} \times \mathcal{L}); \hookrightarrow)$ *for* $\mathsf{Comp}$ *with an equivariant ternary relation,* $\multimap$, *on* $\mathcal{T}$ *such that if* $T_1 \stackrel{T_2}{\multimap} T_3$ *then for any* $C_1 \mathbin{⨾} T_1$ *and* $C_2 \mathbin{⨾} T_2$ *such that*

$\sup(C_1) \cap \sup(C_2) \subseteq \sup(T_1)$, *the state* $C_1|C_2$ *is well-defined, has type* $T_3$ *and satisfies the following* interaction conditions*:*

1. *If* $C_1 \xrightarrow{pl} C'$ *and* $C_2 \xrightarrow{\overline{pl}} C_2'$ *then* $T_1 \overset{pl}{\hookrightarrow} T_1'$ *and* $T_2 \overset{\overline{pl}}{\hookrightarrow} T_2'$ *such that* $T_1' \overset{T_2'}{\multimap} T_3$.
2. *If* $C_2 \xrightarrow{a} C_2'$ *and* $T_3 \overset{a}{\hookrightarrow} T_3'$ *(with* $\sup(T_3') \cap \sup(T_2) \subseteq \sup(T_3)$*) then* $T_2 \overset{a}{\hookrightarrow} T_2'$ *such that* $T_1 \overset{T_2'}{\multimap} T_3'$.
3. *If* $C_1 \xrightarrow{a} C_1'$ *and* $T_3 \overset{a'}{\hookrightarrow} T_3'$ *then* $a \neq a'$.

Informally (1) requires that if $C_1$ and $C_2$ may communicate, then this is permitted by $T_1$ and $T_2$, and (2) and (3) require that the observable actions of $C_1|C_2$ permitted by $T_3$ correspond to actions of $C_2$ permitted by $T_3$. Note that for any $C_1 \mathbin{⁏} T_1$ and $C_2 \mathbin{⁏} T_2$ there exists $C_1' \simeq_{T_1} C_1$ such that $\sup(C_1') \cap \sup(C_2) \subseteq \sup(T_1)$ — i.e. there are no sidechannels of communication between $C_1'$ and $C_2$ — and thus $C_1'|C_2$ is well-defined, has type $T_3$ and satisfies the interaction conditions. Moreover, these are sufficient to establish that typed bisimulation is a congruence with respect to parallel composition with hiding: a result that we will apply to our examples in the rest of the paper.

**Proposition 1.** *If* $C_1 \sim_{T_1} D_1$ *and* $C_2 \sim_{T_2} D_2$ *(and* $\sup(C_1) \cap \sup(C_2), \sup(D_1) \cap \sup(D_2) \subseteq \sup(T_1)$*) where* $T_1 \overset{T_2}{\multimap} T_3$ *then* $C_1|C_2 \sim_{T_3} D_1|D_2$.

*Proof.* We first establish the following renaming property: if $C_1 \mathbin{⁏} T_1 \longrightarrow C_1' \mathbin{⁏} T_1$ then there exists $\pi \in \mathrm{stab}(T_1) \cap \mathrm{stab}(T_2) \cap \mathrm{stab}(T_3)$ such that $C_1|C_2 \mathbin{⁏} T_3 \longrightarrow \pi(C_1')|C_2 \mathbin{⁏} T_3$ — by renaming any fresh names introduced by internal transition so that they are also fresh for $C_2$. Similarly, any internal reduction of $C_2$ corresponds to a reduction of $C_1|C_2$, up to such a renaming.

So suppose $C_1|C_2 \mathbin{⁏} T_3 \xrightarrow{pl} C' \mathbin{⁏} T_3$ (an observable transition). By definition of an interaction structure, and conditions (2) and (3), $C_2 \mathbin{⁏} T_2 \xrightarrow{pl} C_2' \mathbin{⁏} T_3'$ such that $T_1 \overset{T_2'}{\multimap} T_3'$. By assumption, there exists $D_2' \simeq_{T_2} D_2$ such that $D_2' \mathbin{⁏} T_2 \Longrightarrow D_2'' \xrightarrow{a} D_2''' \mathbin{⁏} T_2' \Longrightarrow D_2'''' \mathbin{⁏} T_2'$ and $D_2'''' \sim_{T_2'} C_2'$ and by the renaming property we may rename any fresh names in this reduction sequence to avoid clashes with $D_1$ — i.e. there exists $\pi \in \mathrm{stab}(T_1) \cap \mathrm{stab}(T_2) \cap \mathrm{stab}(T_3)$ such that:
$D_1|D_2' \mathbin{⁏} T_3 \Longrightarrow D_1|\pi(D_2'') \xrightarrow{\pi(a)} D_1|\pi(D_2''') \mathbin{⁏} T_3' \Longrightarrow D_1|\pi(D_2'''') \mathbin{⁏} T_3'$, and hence $\pi^{-1}(D_1)|\pi^{-1}(D_2') \mathbin{⁏} T_3 \overset{pl}{\Longrightarrow} \pi^{-1}(D_1)|D_2''''$ as required (since bisimilarity is closed under permutation of internal names).

If $C_1|C_2 \mathbin{⁏} T_3$ performs an *internal* action then this is either an internal action of $C_1 \mathbin{⁏} T_1$ or $C_2 \mathbin{⁏} T_2$, which is similar to the observable case, or else $C_1 \xrightarrow{pl} C_1'$ and $C_2 \xrightarrow{\overline{pl}} C_2'$ — so that $C_1|C_2$ performs the internal action $l$. Then by interaction condition (1), $T_1 \overset{pl}{\hookrightarrow} T_1'$ and $T_2 \overset{\overline{pl}}{\hookrightarrow} T_2'$ such that $T_1' \overset{T_2'}{\multimap} T_3$. So since $C_1 \sim_{T_1} D_1$ and $C_2 \sim_{T_2} D_2$, there exist $D_1' \sim_{T_1} D_1$ and $D_2' \sim_{T_2} D_2$ such that $D_1' \mathbin{⁏} T_1 \Longrightarrow D_1'' \mathbin{⁏} T_1 \xrightarrow{pl} D_1''' \mathbin{⁏} T_1' \Longrightarrow D_1'''' \mathbin{⁏} T_1'$ and $D_2' \mathbin{⁏} T_2 \Longrightarrow D_2'' \mathbin{⁏} T_2 \xrightarrow{\overline{pl}} D_2''' \mathbin{⁏} T_2' \Longrightarrow D_2'''' \mathbin{⁏} T_1'$ where $C_1' \sim_{T_1'} D_1''''$ and $C_2' \sim_{T_2'} D_2''''$. So using the renaming property we may obtain $\pi \in \mathrm{stab}(T_1) \cap \mathrm{stab}(T_2) \cap \mathrm{stab}(T_3)$ such that $D'_1|D'_2 \,\mathring{\circ}\, T_3 \Longrightarrow \pi(D''_1)|\pi(D''_2) \,\mathring{\circ}\, T_3 \longrightarrow \pi(D'''_1)|\pi(D'''_2) \,\mathring{\circ}\, T_3 \Longrightarrow \pi(D''''_1)|\pi(D'''_2) \,\mathring{\circ}\, T_3$ as required.

## 3 The Lazy $\lambda\mu$-calculus

We now define a typed interaction system giving an interpretation of the (untyped) lazy $\lambda\mu$-calculus — i.e. a direct-style CPS interpretation of lazy functional computation — yielding a novel, direct characterization of normal form bisimulation as typed bisimulation. This acts as a non-trivial example of a typed interaction system (as defined in the previous section) and a stepping stone to the polymorphic typing system for the same underlying language in the next section. First, we define an abstract machine for lazy CPS evaluation, in the form of a nominal LTS in which actions make explicit the calls made by a program to its environment. (Cf the analysis of $\lambda\mu$-calculus by $\pi$-calculus translation in [5].)

**Definition 6.** *The* unnamed *and* named *terms of the untyped $\lambda\mu$-calculus [21] are given (respectively) by the following grammars:*
$t ::= \; x \mid \lambda x.t \mid tt \mid \mu\alpha.M$
$M ::= [\alpha]t$

We equip the set of $\lambda\mu$-terms with a group action by assuming a set $\mathcal{N}$ of distinguished identifiers, partitioned into sorts (infinite subsets) of $\lambda$-variables $(x, y, z, \ldots)$ and $\mu$-variables $(\alpha, \beta, \gamma \ldots)$ and (for later use) type variables $(X, Y, Z, \ldots)$. The group of sort-preserving permutations on $\mathcal{N}$ acts pointwise on expressions (i.e. permuting elements of $\mathcal{N}$ and fixing symbols not in $\mathcal{N}$). We form a nominal set of $\lambda\mu$-terms consisting of the terms in which the free variables are all in $\mathcal{N}$ and those which occur bound (by $\lambda$ or $\mu$) are not, so that the support of a term is its set of free variables.

Based on this syntax, we define the sets of expressions (control terms) which determine the next transition of our abstract machine.

**Definition 7.** Control terms *are given by the grammar:* $\mathcal{A} ::= M \mid V \mid K \mid \bullet$

- *$M$ ranges over the set of $\lambda\mu$* programs *(named terms) — i.e. $M ::= [\alpha]t$.*
- *$V$ ranges over the set of $\lambda\mu$* values *($\lambda$-abstractions) — i.e. $V ::= \lambda x.t$.*
- *$K$ ranges over the set of $\lambda\mu$* continuations *(named contexts with a single hole at head position) — i.e. $K[\bullet] ::= [\alpha]\bullet \mid K[\bullet t]$.*
- *$\bullet$ is the empty context.*

As above we form a nominal set of control terms in which the support of each element is its set of free variables.

**Definition 8.** *An* environment *is a sort-respecting finite partial function $\mathcal{E}$ from $\mathcal{N}$ into the nominal sets of unnamed $\lambda\mu$-terms and continuations. The nominal set of environments has the G-action: $(\pi \cdot \mathcal{E})(a) = \pi \cdot (\mathcal{E}(\pi^{-1} \cdot a))$.*

Direct-style CPS evaluation of a program in an environment proceeds as follows:

- A variable inside a continuation $(\mathcal{E}; K[x])$ fetches the term bound to $x$ and names it with a fresh $\mu$-variable which is bound to $K$.
- A $\beta$-redex inside a continuation $(\mathcal{E}; K[\lambda x.t\, s])$ binds $s$ to a fresh $\lambda$-variable $y$ and $K$ to a fresh $\mu$-variable $\alpha$ and evaluates $[\alpha]t[y/x]$.
- A $\mu$-abstraction inside a continuation $(\mathcal{E}; K[\mu\alpha.M)$ binds $K$ to $\beta$ and evaluates $M[\beta/\alpha]$.
- A named value $(\mathcal{E}; [\alpha]V)$ calls the continuation bound to $\alpha$ with $V$.

These transitions are labelled with actions of the form $a\langle \overrightarrow{b} \rangle$, where $a$ is the variable called (if any) and $\overrightarrow{b}$ are the fresh variables created (if any). Except for $\mu$-abstraction reduction, each of these evaluation rules decomposes into a complementary pair of input and output rules corresponding to the behaviour of the active (or "positive") part of the program and, a passive (or "negative" part). This decomposition is made precise in Definition 10 (parallel composition for configurations).

**Definition 9.** *The nominal labelled transition system* $\mathsf{Comp}_{\lambda\mu}$ *is defined:*

- States *are pairs* $(\mathcal{E}; \mathcal{A})$*, where* $\mathcal{E}$ *is an environment and* $\mathcal{A}$ *is a control term.*
- *The set of* actions *is* $\mathcal{L} \cup (\{+,-\} \times \mathcal{L})$*, where* $\mathcal{L}$ *is the nominal set of* labels

$$\bigcup_{x,\alpha \in \mathcal{N}_\lambda \times \mathcal{N}_\mu} \{\alpha, x\langle\alpha\rangle, \langle\alpha, x\rangle, \langle\alpha\rangle\}$$

- *The* transitions *are given in Table 1. By convention, a variable name mentioned on the right of a rule but not the left is assumed not to occur there.*

The *polarity* of a state is positive if the control term is a program or continuation, and negative if it is a value or the empty context (we write $V_\bullet$ for a passive term of either kind). Unpolarized transitions send positive states to positive states. Except for $\mu$-abstraction reduction, each corresponds to complementary, positive and negative transitions, which send positive states to negative states and vice-versa.

$$(\mathcal{E}[\alpha \mapsto K]; [\alpha]V_\bullet) \xrightarrow{\alpha} (\mathcal{E}; K[V_\bullet])$$
$$(\mathcal{E}; K[(\lambda x.s)\, t]) \xrightarrow{\langle y,\alpha\rangle} (\mathcal{E}, (y \mapsto t), (\alpha \mapsto K); [\alpha]s[y/x])$$
$$(\mathcal{E}[x \mapsto t]; K[x]) \xrightarrow{x\langle\alpha\rangle} (\mathcal{E}, (\alpha \mapsto K); [\alpha]t)$$
$$(\mathcal{E}; K[\mu\alpha.M]) \xrightarrow{\langle\beta\rangle} (\mathcal{E}, (\beta \mapsto K); M[\beta/\alpha])$$

| | |
|---|---|
| $(\mathcal{E}; [\alpha]V_\bullet) \xrightarrow{+\alpha} (\mathcal{E}; V_\bullet)$ | $(\mathcal{E}[\alpha \mapsto K]; V_\bullet) \xrightarrow{-\alpha} (\mathcal{E}; K[V_\bullet])$ |
| $(\mathcal{E}; K[\bullet\, t]) \xrightarrow{+\langle y,\alpha\rangle} (\mathcal{E}, (y \mapsto t), (\alpha \mapsto K); \bullet)$ | $(\mathcal{E}; \lambda x.t) \xrightarrow{-\langle y,\alpha\rangle} (\mathcal{E}; [\alpha]t[y/x])$ |
| $(\mathcal{E}; K[x]) \xrightarrow{+x\langle\alpha\rangle} (\mathcal{E}, (\alpha \mapsto K); \bullet)$ | $(\mathcal{E}[x \mapsto t]; \bullet) \xrightarrow{-x\langle\alpha\rangle} (\mathcal{E}; [\alpha]t)$ |

Table 1: Abstract machine for CPS evaluation of lazy $\lambda\mu$-calculus

$$\begin{array}{cc}
(_; \lambda f.f\,\lambda x.x) & (_; [\alpha] \bullet \lambda y.y) \\
\downarrow {\scriptstyle -\langle g,\beta\rangle} & \downarrow {\scriptstyle +\langle g,\beta\rangle} \\
(_; [\beta]g\,\lambda x.x) & ((\beta \mapsto [\alpha]\bullet), (g \mapsto \lambda y.y); \bullet) \\
\downarrow {\scriptstyle +g\langle\gamma\rangle} & \downarrow {\scriptstyle -g\langle\gamma\rangle} \\
((\gamma \mapsto [\beta]\bullet\ \lambda x.x); \bullet) & ((\beta \mapsto [\alpha]\bullet), (g \mapsto \lambda y.y); [\gamma]\lambda y.y) \\
\downarrow {\scriptstyle -\gamma} & \downarrow {\scriptstyle +\gamma} \\
((\gamma \mapsto [\beta] \bullet \lambda x.x); [\beta]\bullet\ \lambda x.x) & ((\beta \mapsto [\alpha]\bullet), (g \mapsto \lambda y.y); \lambda y.y) \\
\downarrow {\scriptstyle +\langle z,\delta\rangle} & \downarrow {\scriptstyle -\langle z,\delta\rangle} \\
(\gamma \mapsto [\beta]\bullet\ \lambda x.x), (z \mapsto \lambda x.x), (\delta \mapsto [\beta]\bullet); \bullet) & ((\beta \mapsto [\alpha]\bullet), (g \mapsto \lambda y.y); [\delta]z) \\
\downarrow {\scriptstyle -z\langle\epsilon\rangle} & \downarrow {\scriptstyle +z\langle\epsilon\rangle} \\
(\gamma \mapsto [\beta]\bullet\ \lambda x.x), (z \mapsto \lambda x.x), (\delta \mapsto [\beta]\bullet); [\epsilon]\lambda x.x) & ((\beta \mapsto [\alpha]\bullet), (g \mapsto \lambda y.y), (\epsilon \mapsto [\delta]\bullet); \bullet) \\
\downarrow {\scriptstyle +\epsilon} & \downarrow {\scriptstyle -\epsilon} \\
(\gamma \mapsto [\beta]\bullet\ \lambda x.x), (z \mapsto \lambda x.x), (\delta \mapsto [\beta]\bullet); \lambda x.x) & ((\beta \mapsto [\alpha]\bullet), (g \mapsto \lambda y.y), (\epsilon \mapsto [\delta]\bullet); [\delta]\bullet) \\
\downarrow {\scriptstyle -\delta} & \downarrow {\scriptstyle +\delta} \\
(\gamma \mapsto [\beta]\bullet\ \lambda x.x), (z \mapsto \lambda xy.x), (\delta \mapsto [\beta]\bullet); [\beta]\lambda x.x) & ((\beta \mapsto [\alpha]\bullet), (g \mapsto \lambda y.y), (\epsilon \mapsto [\delta]\bullet); \bullet) \\
\downarrow {\scriptstyle +\beta} & \downarrow {\scriptstyle -\beta} \\
(\gamma \mapsto [\beta]\bullet\ \lambda x.x), (z \mapsto \lambda x.x), (\delta \mapsto [\beta]\bullet); \lambda x.x) & ((\beta \mapsto [\alpha]\bullet), (g \mapsto \lambda y.y), (\epsilon \mapsto [\delta]\bullet); [\alpha]\bullet)
\end{array}$$

Fig. 1: Example traces evaluating $[\alpha](\lambda f.f\,\lambda x.x)\,\lambda y.y$

To define an *interaction structure* on $\mathsf{Comp}_{\lambda\mu}$ (Definition 4) we require a parallel composition operation on configurations.

**Definition 10.** *[Parallel Composition] On control terms, let $|$ be the (least) partial operation such that $\mathcal{A}|\bullet = \bullet|\mathcal{A} = \mathcal{A}$ and $K|V = V|K = K[V]$.*
*Given configurations $C_1 = (\mathcal{E}_1; \mathcal{A}_1)$ and $C_2 = (\mathcal{E}_2; \mathcal{A}_2)$ let $C_1|C_2 \triangleq (\mathcal{E}_1 \cup \mathcal{E}_2; \mathcal{A}_1|\mathcal{A}_2)$, provided $\mathsf{dom}(\mathcal{E}) \cap \mathsf{dom}(\mathcal{E}) = \varnothing$ and $\mathcal{A}_1|\mathcal{A}_2$ is well-defined. ($C_1|C_2$ is undefined, otherwise.)*

By inspection of the transitions in Table 1, we may see that $C_1|C_2$ has precisely the transitions of $C_1$ or $C_2$ (provided any fresh names are fresh for $C_1|C_2$), together with internal transitions arising from communication between $C_1$ and $C_2$. Therefore we have an interaction structure according to Definition 4. Figure 1 gives an illustrative example: the evaluation of $[\alpha](\lambda f.f\,\lambda x.x)\,\lambda y.y$ — which is the parallel composition $(\lambda f.f\,\lambda x.x)|([\alpha] \bullet \lambda y.y)$ — to $[\alpha]\lambda x.x$.

## 3.1 A Typing System

We now define a basic typing system for configurations which records minimal information about the control term (whether it is a program, value, continuation or empty context) but captures a more significant property of environments — acyclicity. This has practical relevance for memory management, but its immediate significance is that the second order typing in the next section relies on the fact that an acyclic environment may be contracted into a *valuation* by iteratively replacing variables bound in the environment until none occur as free variables.

**Definition 11.** *Given a nominal environment $\mathcal{E}$, define the binary relation on $\mathcal{N}$: $a \ll_{\mathcal{E}} b$ if $a \in \sup(\mathcal{E}(b))$ and let $\ll_{\mathcal{E}}^*$ be its transitive closure. Say that $\mathcal{E}$ is a* pre-valuation *(i.e. acyclic) if this is a strict partial order — i.e. $a \not\ll^* a$ for all $a \in \mathcal{N}$. $\mathcal{E}$ is a* valuation *if $\ll_{\mathcal{E}} = \ll_{\mathcal{E}}^*$ — i.e. $\sup(\mathcal{E}(a)) \cap \mathsf{dom}(\mathcal{E}) = \varnothing$ for all $a \in \mathsf{dom}(\mathcal{E})$.*

We assume a closure operation which takes an expression $e$ and pre-valuation $\mathcal{E}$ to an expression $\mathcal{E}(e)$ obtained by replacing each atom $a \in \mathsf{dom}(\mathcal{E})$ with $\mathcal{E}(a)$ in $e$, having the property that $\sup(\mathcal{E}(e)) \cap \mathsf{dom}(\mathcal{E}) = \bigcup\{\sup(\mathcal{E}(a)) \mid a \in \sup(e) \cap \mathsf{dom}(\mathcal{E})\}$.

**Lemma 1.** *For any pre-valuation $\mathcal{E}$ there is a unique valuation $\mathcal{E}^*$ such that $\mathcal{E}^*(\mathcal{E}(e)) = \mathcal{E}^*(e)$ for all expressions $e$.*

*Proof.* Defining $\mathcal{E}^i$ by $\mathcal{E}^{i+1}(a) = \mathcal{E}^i(\mathcal{E}(a))$, the $\mathcal{E}^i$ form a chain of pre-evaluations such that the $\ll_{\mathcal{E}}$ downward closure of $\bigcup\{\sup(\mathcal{E}^i(a)) \cap \mathsf{dom}(\mathcal{E}) \mid a \in \mathsf{dom}(\mathcal{E})\}$ is empty or strictly decreasing, and thus is empty for some $k$ — i.e. $\mathcal{E}^k$ is a pre-valuation and thus $\mathcal{E}^k(\mathcal{E}(a)) = \mathcal{E}(\mathcal{E}^k(a)) = \mathcal{E}^k(a)$ for all $a \in \mathsf{dom}(\mathcal{E})$, and so $\mathcal{E}^*(\mathcal{E}(e)) = \mathcal{E}^*(e)$ for all expressions $e$. If $\mathcal{E}^*(e) = \mathcal{E}^*(\mathcal{E}(e))$ for all expressions $e$, then $\mathcal{E}^*(e) = \mathcal{E}^*(\mathcal{E}^k(e)) = \mathcal{E}^k(e)$ for all $e$.

**Definition 12.** *The* basic *types for control terms are tuples $\Gamma \vdash \tau; \Delta$ where $\tau \in \{\top, \bot\}$ and $\Gamma, \Delta$ are non-repeating sequences — i.e. totally ordered finite sets — of $\lambda$ and $\mu$ variables in $\mathcal{N}$, respectively.*

*A control term $\mathcal{A}$ is well-typed with $\Gamma \vdash \tau; \Delta$ if $FV(\mathcal{A}) \subseteq \Gamma \cup \Delta$ and $\tau = \top$ if and only if $\mathcal{A}$ is a value or continuation. Basic types form a nominal set with the evident pointwise $G$-action.*

Configurations are typed with polarized versions of these types. Given a polarized context (non-repeating sequence of polarized variables) $\Gamma = p_1x_1, \ldots, p_nx_n$ we write $|\Gamma|$ for the unpolarized context $x_1, \ldots, x_n$, $\overline{\Gamma}$ for the polarized context $\overline{p_1}x_1, \ldots, \overline{p_n}x_n$, and $\Gamma^p$ for the (unpolarized) restriction of $\Gamma$ to $p$-polarized elements.

**Definition 13.** *The nominal LTS $\mathsf{Ty}_{\lambda\mu}$ of basic $\lambda\mu$ configuration types:*

- States *are polarized configuration types — triples $\Gamma \vdash p\tau; \Delta$, where $p\tau \in \{+, -\} \times \{\top, \bot\}$ and $\Gamma$ and $\Delta$ are polarized contexts of $\lambda$ and $\mu$ variables in $\mathcal{N}$.*
- Actions *are the polarized actions of $\mathsf{Comp}_{\lambda\mu}$ — Obs $= \{+, -\} \times \mathcal{L}$*
- Transitions *are given by the rules in Table 2.*

We now define a typing relation from configurations to types. Let $\Gamma$ be a polarized context. A pre-valuation for $\Gamma$ is a pre-valuation $\mathcal{E}$ such that $\Gamma^+ \subseteq \mathsf{dom}(\mathcal{E})$, $\sup(\mathcal{E}(a)) \subseteq \mathsf{dom}(\mathcal{E}) \cup \Gamma^-$ for every $a \in \mathsf{dom}(\mathcal{E})$, and if $a, b \in \Gamma$ and $a \ll_{\mathcal{E}}^* b$ then $a <_{\Gamma} b$. Observe that if $\mathcal{E}$ is a pre-valuation for $\Gamma$, then $\mathcal{E}^*$ is a valuation for $\Gamma$ such that for all $a \in \Gamma^+$, $FV(\mathcal{E}^*(a)) \subseteq \Gamma^-$.

$$\begin{array}{lcl} \Gamma \vdash p\top;\Delta & \stackrel{p\langle x,\alpha\rangle}{\hookrightarrow} & \Gamma, px \vdash \overline{p}\bot;\Delta, p\alpha \\ \Gamma[\overline{p}x] \vdash p\bot;\Delta & \stackrel{px\langle\alpha\rangle}{\hookrightarrow} & \Gamma \vdash \overline{p}\bot;\Delta, p\alpha \\ \Gamma \vdash p\top;\Delta[\overline{p}\alpha] & \stackrel{p\alpha}{\hookrightarrow} & \Gamma \vdash \overline{p}\bot;\Delta \\ \Gamma \vdash p\bot;\Delta[\overline{p}\alpha] & \stackrel{p\alpha}{\hookrightarrow} & \Gamma \vdash \overline{p}\top;\Delta \end{array}$$

Table 2: Transitions of basic configuration types

**Definition 14 ($\lambda\mu$ Typing Relation).** $(\mathcal{E};\mathcal{A}) \mathbin{⸬} (\Gamma \vdash p\tau;\Delta)$ *if* $\mathsf{pol}(\mathcal{E};\mathcal{A}) = p$ *and* $\mathcal{E}$ *is a pre-valuation for* $\Gamma \cup \Delta$ *such that* $\Gamma^- \vdash \mathcal{E}^*(\mathcal{A}) : \tau;\Delta^-$*, and for each* $x \in \Gamma^+$*,* $\Gamma^- \vdash \mathcal{E}^*(x) : \top;\Delta^-$ *and each* $\alpha \in \Delta^+$*,* $\Gamma^- \vdash \mathcal{E}^*(\alpha) : \top;\Delta^-$*.*

It is straightforward to check that this satisfies the subject reduction properties and thus defines a type system for $\mathsf{Comp}_{\lambda\mu}$.

*Remark 1.* We may apply a second constraint via our type system: *local control flow* — that continuations are called according to a LIFO discipline and thus may be stored on a stack (in game semantic terms, the *well-bracketing condition*). Evaluation of $\lambda$-terms by internal (and positive) transitions naturally satisfies this property — we can use types to ensure that the environment also does so.

**Definition 15.** *A configuration type* $\Gamma \vdash p\tau;\Delta$ *satisfies the local control condition if the polarities of* $\mu$*-variables in* $\Delta$ *are alternating, and the polarity of the last element of* $\Delta$ *(if any) is* $\overline{p}$*.*

Transitions for local control types are given by refining the rules for calling a continuation to enforce stack discipline:

$$\Gamma \vdash p\top;\Delta,\overline{p}\alpha \xrightarrow{p\alpha} \Gamma \vdash \overline{p}\bot;\Delta$$
$$\Gamma \vdash p\bot;\Delta,\overline{p}\alpha \xrightarrow{p\alpha} \Gamma \vdash \overline{p}\top;\Delta$$

Subject reduction holds with respect to $\lambda$-configurations (in which the control term, and all terms and continuations in the environment, contain no $\mu$-abstractions).

### 3.2 A Typed Interaction Structure

We now define an arrow relation, allowing a characterization of parallel composition with hiding for acyclic configurations. (Acyclicity is not preserved by union of environments in general, so the typing rules give a useful way of identifying pairs of configurations for which it does hold.)

**Definition 16.** *The* arrow relation *on configurations* $T_i = \Gamma_i \vdash p\tau_i;\Delta_i$ *is defined pointwise —* $T_1 \stackrel{T_2}{\multimap} T_3$ *if* $\Gamma_1 \stackrel{\Gamma_2}{\multimap} \Gamma_3$*,* $\Delta_1 \stackrel{\Delta_2}{\multimap} \Delta_3$*, and* $p\tau_1 \stackrel{p\tau_2}{\multimap} p\tau_3$ *— where*

– *For any polarized contexts,* $\Sigma_1 \stackrel{\Sigma_2}{\multimap} \Sigma_3$ *if* $\Sigma_1$ *and* $\Sigma_3$ *have disjoint underlying sets of elements and* $\Sigma_2$ *is an interleaving of* $\overline{\Sigma_1}$ *and* $\Sigma_3$*.*

– $p\tau_1 \overset{p\tau_2}{\multimap} p\tau_3$ *iff* $p\tau_1 = -\bot$ *and* $p\tau_2 = p\tau_3$ *or* $p\tau_3 = +\bot$ *and* $p\tau_2 = \overline{p}\tau_1$.

It remains to show that this satisfies Definition 5.

**Proposition 2.** $(\mathsf{Ty}_{\lambda\mu}, \multimap)$ *is a well-defined typing system for* $(\mathsf{Comp}_{\lambda\mu}, |)$.

*Proof.* Given $C_1 = (\mathcal{E}_1; \mathcal{A}_1)$ and $C_2 = (\mathcal{E}_2; \mathcal{A}_2)$, suppose $C_1 : T_1$, $C_2 : T_2$ and $\sup(C_1) \cap \sup(C_2) \subseteq \sup(T_1) = |\Gamma_1| \cup |\Delta_1|$:

– $\mathcal{A}_1|\mathcal{A}_2$ is well-defined, and has type $\tau_3$, since either $\mathcal{A}_1 : -\bot$ (i.e. $\mathcal{A}_1 = \bullet$) and so $\mathcal{A}_1|\mathcal{A}_2 : \tau_2$, or $\mathcal{A}_1$ and $\mathcal{A}_2$ have complementary types, and so $\mathcal{A}_1|\mathcal{A}_2 : +\bot$ (i.e. they are a term and context which fit together to give a program).
– $\mathcal{E}_1 \cup \mathcal{E}_2$ is a pre-valuation, since the directed graph $(\ll_{\mathcal{E}_1} \cup \ll_{\mathcal{E}_2})$ is acyclic. (Any cycle in this graph would have to contain vertices from both $\ll_{\mathcal{E}_1}$ and $\ll_{\mathcal{E}_2}$, since both fragments are acyclic. Any path which enters and leaves one fragment must begin and end on points which are ordered by $\Gamma \cup \Delta$ and so composing such paths cannot lead to a cycle.)

Moreover, it is straightforward to verify that the interaction conditions are satisfied and that we therefore have a typed interaction structure.

Thus, by Proposition 1, typed bisimilarity is preserved by parallel composition plus hiding.

**Proposition 3.** *If* $C_1 \sim_{T_1} D_1$, $C_2 \sim_{T_2} D_2$ *and* $T_1 \overset{T_2}{\multimap} T_3$ *then* $C_1|C_2 \sim_{T_3} D_1|D_2$.

It immediately follows that (for example) bisimilarity of values is preserved by placing them inside the same continuation — i.e. if $(\_; v)$ and $(\_; v')$ are bisimilar at type $\Gamma \vdash -\top; \Delta$ then $(\_; K[v])$ and $(\_; K[v'])$ are bisimilar at type $\Gamma \vdash +\bot; \Delta$. Moreover, if typed bisimilarity is extended to an equivalence on all $\lambda\mu$-terms — $s \sim_{\Gamma;\Delta} t$ if $(\_; [\alpha]s) \sim_{-\Gamma \vdash +\bot; -\Delta, -\alpha} (\_; [\alpha]t)$, for $\alpha \notin \Delta$ — we may use Proposition 3 to show that if $s \sim_{\Gamma;\Delta} t$ then for any compatible context, $C[t] \sim_{\Gamma;\Delta} C[t']$.

## 4 A Polymorphic Type System

In this section we describe a more restrictive and informative typing system for the interaction structure of $\lambda\mu$ configurations. This yields a model of the lazy $\lambda\mu 2$-calculus — i.e. lazy $\lambda\mu$-calculus with polymorphic (second-order) Curry-style typing, which we now describe.

In order to fit such a type system to a semantics of lazy evaluation to weak head-normal form, we combine $\lambda$-abstraction and application with abstraction and instantiation of finite sequences of type variables — i.e. function types take the form $\forall(X_1 \ldots X_n).\sigma \to \tau$, where $X_1 \ldots X_n$ is a finite, non-repeating sequence of type variables. The judgments $\Theta \vdash \tau$ ($\tau$ is a well-formed type over the context of type-variables $\Theta$) are derived according to the rules:

$$\frac{}{\Theta, X, \Theta' \vdash X} \qquad \frac{\Theta, X_1, \ldots, X_n \vdash \sigma \quad \Theta, X_1, \ldots, X_n \vdash \tau}{\Theta \vdash \forall(X_1 \ldots X_n).\sigma \to \tau}$$

Typing judgments are given with respect to an *equational context* (finite sequence of equations between types). These contexts play a key role in defining states in our LTS of types — they record constraints that type-instantiations must satisfy. For example, if a continuation $K$ (with a hole) of type $\sigma$ is called with an argument $v$ of type $\tau$ then the type variables in $\sigma$ and $\tau$ must have been instantiated so as to make these types equal. Formally, we define the judgment $\Theta \vdash \Xi$ ($\Xi$ is a well-formed equational context over $\Theta$) as follows:

$$\frac{}{\Theta \vdash \_} \qquad \frac{\Theta \vdash \Xi \quad \Theta \vdash \sigma \quad \Theta \vdash \tau}{\Theta \vdash \Xi, \sigma = \tau}$$

Type equality judgments with respect to an equational context, of the form $\Theta; \Xi \vdash \sigma = \tau$ (where $\Theta \vdash \Xi, \sigma, \tau$) are derived according to the rules:

$$\frac{}{\Theta;\Xi[\sigma=\tau]\vdash\sigma=\tau} \qquad \frac{}{\Theta;\Xi\vdash\tau=\tau} \qquad \frac{\Theta;\Xi\vdash\rho=\tau \quad \Theta;\Xi\vdash\sigma=\tau}{\Theta;\Xi\vdash\rho=\sigma}$$

$$\frac{\Theta;\Xi\vdash\forall\overrightarrow{X}.\sigma\to\tau=\forall\overrightarrow{X}.\sigma'\to\tau'}{\Theta,\overrightarrow{X};\Xi\vdash\sigma=\sigma'} \quad \frac{\Theta;\Xi\vdash\forall\overrightarrow{X}.\sigma\to\tau=\forall\overrightarrow{X}.\sigma'\to\tau'}{\Theta,\overrightarrow{X}\vdash\tau=\tau'} \quad \frac{\Theta,\overrightarrow{X};\Xi\vdash\sigma=\sigma' \quad \Theta,\overrightarrow{X}\vdash\tau=\tau'}{\Theta;\Xi\vdash\forall\overrightarrow{X}.\sigma\to\tau=\forall\overrightarrow{X}.\sigma'\to\tau'}$$

A valuation $\mathcal{V}$ for $\Theta$ *satisfies* an equational context $\Theta \vdash \sigma_1 = \tau_1, \ldots, \sigma_n = \tau_n$ if $\mathcal{V}(\sigma_i) \equiv \mathcal{V}(\tau_i)$ for each $i \leq n$.

**Lemma 2.** *$\Theta; \Xi \vdash \sigma = \tau$ if and only if for all valuations $\mathcal{V}$ which satisfy $\Xi$, $\mathcal{V}(\sigma) \equiv \mathcal{V}(\tau)$.*

A $\lambda\mu 2$ type-in-context is a tuple $\Theta; \Xi; \Gamma \vdash \tau; \Delta$, where $\Theta$ is a context of type variables and $\Xi$ is an equational context, $\tau$ is a $\lambda\mu 2$-type (or $\bot$) and $\Gamma$ and $\Delta$ are (respectively) sequences of $\lambda$-variables and $\mu$-variables and their types (all over $\Theta$). Assigning this type to a term may be understood as asserting that "for any valuation $\mathcal{V}$ of the type-variables in $\Theta$ which satisfies $\Xi$, the judgement $\mathcal{V}(\Gamma) \vdash t : \mathcal{V}(\tau); \mathcal{V}(\Delta)$ is valid". So, for example, $X, Y; Y = X \to X; \_ \vdash \lambda x.x : Y;$ is derivable according to the rules in Table 3. Note that there are no rules for introducing or discharging equational assumptions — they will be generated by the transitions of the LTS — so the terms of type $\Theta; \_; \Gamma \vdash t : \tau; \Delta$ are precisely those derivable in second-order $\lambda\mu$-calulus without type equality judgments.

$$\frac{}{\Theta;\Xi;\Gamma[x{:}\tau]\vdash x{:}\tau;\Delta} \qquad \frac{\Theta;\Xi;\Gamma\vdash t{:}\sigma;\Delta \quad \Theta;\Xi\vdash\sigma=\tau}{\Theta;\Xi;\Gamma\vdash t{:}\tau;\Delta} \qquad \frac{\Theta,X_1{:}\kappa,\ldots,X_n{:}\kappa_n;\Xi;\Gamma,x{:}\sigma\vdash t{:}\tau;\Delta}{\Theta;\Xi;\Gamma\vdash\lambda x.t{:}\forall X_1\ldots X_n.(\sigma\to\tau);\Delta}$$

$$\frac{\Theta;\Xi;\Gamma\vdash t{:}\forall X_1\ldots X_n.\sigma\to\tau;\Delta \quad \Theta\vdash\rho_1,\ldots,\rho_n \quad \Theta;\Xi;\Gamma\vdash s{:}\sigma[\rho_1/X_1\ldots\rho_n/X_n];\Delta}{\Theta;\Xi;\Gamma\vdash t\,s{:}\tau[\rho_1/X_1\ldots\rho_n/X_n];\Delta}$$

$$\frac{\Theta;\Xi;\Gamma\vdash t{:}\tau;\Delta[\alpha{:}\tau]}{\Theta;\Xi;\Gamma\vdash[\alpha]t{:}\bot;\Delta} \qquad \frac{\Theta;\Xi;\Gamma\vdash M{:}\bot;\Delta,\alpha{:}\tau}{\Theta;\Xi;\Gamma\vdash\mu x.M{:}\tau;\Delta}$$

Table 3: Typing Judgments for the lazy $\lambda\mu 2$-Calculus

### 4.1 Second-Order Configuration Types

We now define a second-order typing system for the interaction structure $\mathsf{Comp}_{\lambda\mu}$ of $\lambda\mu$ configurations. Its states (second-order configuration types) capture the totality of information about the types of the control term and environment, and the instantiations for type variables by both a program and its environment, which may be inferred by an external observer of their interaction.

**Definition 17.** *A* second-order configuration type *is a polarized* $\lambda\mu 2$ *type-in-context — a tuple* $\Theta; \Xi; \Gamma; \vdash p\tau; \Delta$, *where* $\Theta$ *is a polarized context of type-variables, and* $\Xi$ *is a polarized equational context,* $\Gamma$ *and* $\Delta$ *are polarized contexts of typed* $\lambda$ *and* $\mu$ *variables and* $p\tau$ *is a polarized* $\lambda\mu 2$*-type (or* $\bot$*), all over* $\Theta$ *.*

We place a further constraint — "polarized satisfiability" — on the configuration types which are permitted as states. This requires that their equational contexts can actually be satisfied by a program and environment successively instantiating type variables quantified positively and negatively (respectively), without knowing the types instantiated by the counterparty.

**Definition 18.** *A pre-valuation* $\mathcal{V}$ *for a polarized context of type variables* $\Theta$ positively satisfies *the polarized equational context* $\Theta \vdash \Xi$ *(written* $\mathcal{V} \vDash_{\Theta} \Xi$*) if for any pre-valuation* $\mathcal{W}$ *for* $\overline{\Theta}$, *the first formula in* $\Xi$ not *satisfied by the valuation* $(\mathcal{V} \cup \mathcal{W})^*$ *for* $|\Theta|$ *(if any) is negative.* $\Theta \vdash \Xi$ *is (polarized)* satisfiable *if* $\Xi \vdash \Theta$ *and* $\overline{\Theta} \vdash \overline{\Xi}$ *are both positively satisfiable. Note that this implies that the underlying context* $|\Theta| \vdash |\Xi|$ *is satisfiable.*

Determining whether a polarized context is satisfiable is equivalent to a series of *conditional (first-order) unification* problems: these can be solved using the algorithm for first-order unification [11]. We place an equivalence relation on configuration types (cf. structural congruence of processes), allowing the principal type to be replaced by any of the (finitely many) types to which it is equivalent under $\Xi$.

**Definition 19.** $(\Theta; \Xi; \Gamma \vdash p\tau; \Delta) \eqsim (\Theta; \Xi; \Gamma \vdash p\tau'; \Delta)$ *if* $\Theta; \Xi \vdash \tau = \tau'$.

The (bipartite, nominal) LTS $\mathsf{Ty}_{\lambda\mu 2}$ of $\lambda\mu 2$ is defined:

– States are $\eqsim$-classes of satisfiable configuration types $\Theta; \Xi; \Gamma \vdash p\tau; \Delta$.
– *Actions* are polarized actions of $\mathsf{Comp}_{\lambda\mu}$: $Obs = \{+, -\} \times \mathcal{L}$.
– *Transitions* are given by the rules in Table 4.

To define a typing relation between configurations and $\lambda\mu 2$-configuration types, we first define typing judgements $\Theta; \Xi; \Gamma \vdash \mathcal{A} : \tau; \Delta$ for control terms. In the case of programs and values, these are as derived according to the rules in Table 3. For continuations, the rules

$$\frac{}{\Theta;\Xi;\Gamma\vdash[\alpha]\bullet:\tau;\Delta[\alpha:\tau]} \qquad \frac{\Theta;\Xi;\Gamma\vdash K:\tau[\rho_1/X_1...\rho_n/X_n];\Delta \qquad \Theta;\Xi;\Gamma\vdash s:\sigma[\rho_1/X_1...\rho_n/X_n]}{\Theta;\Xi;\Gamma\vdash K[\bullet\, s]:\forall X_1...X_n.\sigma\to\tau;\Delta}$$

are equivalent to typing $\Theta; \Xi; \Gamma \vdash K : \tau; \Delta$ if $\Theta; \Xi; \Gamma, \bullet : \tau \vdash K[\bullet] : \bot; \Delta$. The empty context has type $\bot$ in any well-formed context.

$$\begin{array}{lcl}
\Theta;\Xi;\Gamma \vdash p\forall X_1 \ldots X_n.\sigma \to \tau;\Delta & \overset{p\langle x,\alpha\rangle}{\hookrightarrow} & \Theta, pX_1,\ldots,pX_n;\Xi;\Gamma, px:\sigma \vdash \overline{p}\bot;\Delta, p\alpha:\tau \\
\Theta;\Xi;\Gamma[\overline{p}x:\tau];p\bot & \overset{px\langle\alpha\rangle}{\hookrightarrow} & \Theta;\Xi;\Gamma \vdash \overline{p}\bot;\Delta, p\alpha:\tau \\
\Theta;Xi;\Gamma \vdash p\bot;\Delta[\overline{p}\alpha:\tau] & \overset{p\alpha}{\hookrightarrow} & \Theta;\Xi;\Gamma \vdash \overline{p}\tau;\Delta \\
\Theta;\Xi;\Gamma \vdash p\sigma;\Delta[\overline{p}\alpha:\tau] & \overset{p\alpha}{\hookrightarrow} & \Theta;\Xi, p(\sigma=\tau);\Gamma \vdash \overline{p}\bot;\Delta
\end{array}$$

Table 4: Transitions of second-order configuration types

**Definition 20 (Typing Relation).** *Let $\mathcal{V}$ be a valuation for $\Theta$ which positively satisfies $\Xi$, and define $\mathcal{V} \vDash (\mathcal{E};\mathcal{A}) ⨟ \Theta;\Xi;\Gamma \vdash p\tau;\Delta$ if $\mathcal{E}$ is a pre-valuation for $\Gamma,\Delta$, such that $\Theta^-;\mathcal{V}(\Xi^-);\mathcal{V}(\Gamma^-) \vdash \mathcal{E}^*(\mathcal{A})) : \mathcal{V}(\tau);\mathcal{V}(\Delta^-)$ and for each $x:\sigma \in \Gamma^+$, $\Theta^-;\mathcal{V}(\Xi^-);\mathcal{V}(\Gamma^-) \vdash \mathcal{E}^*(x) : \mathcal{V}(\sigma);\mathcal{V}(\Delta^-)$ and each $\alpha:\sigma \in \Delta^+$, $\Theta^-;\mathcal{V}(\Xi^-);\mathcal{V}(\Gamma^-) \vdash \mathcal{E}^*(\alpha):\mathcal{V}(\sigma);\mathcal{V}(\Delta^-)$.*

*Let $C ⨟ T$ if there exists a valuation $\mathcal{V}$ for $\Theta$ such that $\mathcal{V} \vDash C ⨟ T$.*

Note that if $C ⨟ T$ and $T \eqsim T'$ then $C ⨟ T'$, so typing is a well-defined relation from configurations to equivalence classes of configuration types.

**Proposition 4.** $(\mathsf{Comp}_{\lambda\mu} ⨟ \mathsf{Ty}_{\lambda\mu2})$ *satisfies the subject reduction property.*

*Proof.* For the observable transitions, this is a straightforward observation that the typing relation is preserved. For internal transitions (specifically, $\beta$ reductions), we use the corresponding subject reduction property for $\lambda\mu2$ substitutions — i.e. if $\Theta;\Xi;\Gamma \vdash K[\lambda x.t\,s]:\bot;\Delta$ then $\Theta;\Xi;\Gamma \vdash K[t[s/x]]:\bot;\Delta$ and if $\Theta;\Xi;\Gamma \vdash K[\mu\alpha.t]:\bot;\Delta$ then $\Theta;\Xi;\Gamma \vdash t[K/\alpha]:\bot;\Delta$.

Figure 2 gives an example illustrating the role of types in constraining behaviour: a trace of the value $\lambda f.f\,v ⨟ \exists X.X$, where $v$ is an arbitrary typable value (recall that $\exists X.X \triangleq \forall Y.(\forall X.X \to Y) \to Y$). Observe that there are no transitions from the the final state — a call to $\gamma$ is not possible because $-Y,+X \vdash -(Y' = X')$ is not negatively satisfiable. In fact, the tree of transitions of $\exists X.X$ branches only on negative transitions (i.e. Opponent moves). It follows that any configuration of this type will have the same set of transitions, and that therefore $\lambda f.f\,\lambda xy.x \sim_{\exists X.X} \lambda f.f\,\lambda xy.y$ as proposed in the introduction.

### 4.2 A Second-Order Typed Interaction Structure

It remains to prove that $\mathsf{Ty}_{\lambda\mu2}$ is a well-defined typing system for the interaction structure on $\mathsf{Comp}_{\lambda\mu}$, and that typed bisimulation is therefore a congruence. We need to establish that the pointwise extension of the arrow relation (Definition 16) to second-order configuration types (i.e. $T_1 \overset{T_2}{\multimap} T_3$ if $\Theta_1 \overset{\Theta_2}{\multimap} \Theta_3$, $\Xi_1 \overset{\Xi_2}{\multimap} \Xi_3$, $\Gamma_1 \overset{\Gamma_2}{\multimap} \Gamma_3$, $\Delta_1 \overset{\Delta_2}{\multimap} \Delta_3$, and $p\tau_1 \overset{p\tau_2}{\multimap} p\tau_3$) satisfies the conditions of Definition 5 — that if $C_1 = (\mathcal{E}_1;\mathcal{A}_1) ⨟ T_1$ and $C_2 = (\mathcal{E}_2;\mathcal{A}_2) ⨟ T_2$, where $T_1 \overset{T_2}{\multimap} T_3$ and

$$
\begin{array}{c}
(\_; \lambda f.f\,v) \fatsemi (\_; \_; \_ \vdash -(\forall X.X \to Y) \to Y; \_) \\
\big\downarrow{\scriptstyle -\langle g,\alpha \rangle} \\
(\_; [\alpha]g\,v) \fatsemi (-Y'; \_; -g : \forall X.X \to Y' \vdash +\bot; -\alpha : Y') \\
\big\downarrow{\scriptstyle +g\langle \beta \rangle} \\
((\beta \mapsto [\alpha] \bullet v); \bullet) \fatsemi (-Y'; \_; -g : \forall X.X \to Y' \vdash -\bot; -\alpha : Y', +\beta : \forall X.X \to Y' \\
\big\downarrow{\scriptstyle -\beta} \\
((\beta \mapsto [\alpha] \bullet v); [\alpha] \bullet v) \fatsemi (-Y'; \_; -g : \forall X.X \to Y' \vdash +\forall X.X \to Y'; -\alpha : Y') \\
\big\downarrow{\scriptstyle +\langle z,\gamma \rangle} \\
((\beta \mapsto [\alpha] \bullet v), (z \mapsto v), (\gamma \mapsto [\alpha]\bullet); \bullet) \fatsemi (-Y', +X'; \_; -g : \forall X.X \to Y', +z : X' \vdash -\bot; -\alpha : Y', +\gamma : Y') \\
\big\downarrow{\scriptstyle -z\langle \delta \rangle} \\
((\beta \mapsto [\alpha] \bullet v), (z \mapsto v), (\gamma \mapsto [\alpha]\bullet); [\delta]v) \fatsemi (-Y', +X'; \_; -g : \forall X.X \to Y', +z : X' \vdash +\bot; -\alpha : Y', +\gamma : Y', -\delta : X') \\
\big\downarrow{\scriptstyle +\delta} \\
((\beta \mapsto [\alpha] \bullet v), (z \mapsto v), (\gamma \mapsto [\alpha]\bullet); v) \fatsemi (-Y', +X'; \_; -g : \forall X.X \to Y', +z : X' \vdash -X'; -\alpha : Y', +\gamma : Y')
\end{array}
$$

Fig. 2: Trace of $\lambda f.f\,v : \exists X.X$

$\sup(C_1) \cap \sup(C_2) \subseteq \sup(T_1)$, then $C_1|C_2$ is well-defined, has type $T_3$ and satisfies the interaction conditions.

By Proposition 2, $C_1|C_2 = (\mathcal{E}_1 \cup \mathcal{E}_2; \mathcal{A}_1|\mathcal{A}_2)$ is a well-defined configuration, and $\mathcal{E} \triangleq \mathcal{E}_1 \cup \mathcal{E}_2$ is a pre-valuation for $\Gamma_3 \cup \Delta_3$. By the assumption that $C_1 \fatsemi T_1$ and $C_2 \fatsemi T_2$, there are valuations $\mathcal{V}_1 \vDash C_1 \fatsemi T_1$ and $\mathcal{V}_2 \vDash C_2 \fatsemi T_2$. Then $\mathcal{V} \triangleq \mathcal{V}_1 \cup \mathcal{V}_2$ is a pre-valuation for $\Theta_3$. To show that $\mathcal{V}^* \vDash C_1|C_2 \fatsemi T_3$, we need to verify that:

**Lemma 3.** *$\mathcal{V}$ positively satisfies $\Xi_3$.*

*Proof.* Let $\mathcal{W}$ be a pre-valuation for $\overline{\Theta_3}$. The first formula in $\Xi_2$ (if any) which is not satisfied by $\mathcal{V} \cup \mathcal{W} = \mathcal{V}_1 \cup \mathcal{V}_2 \cup \mathcal{W}$ cannnot be positive in $\Xi_1$ (positively satisfied by $\mathcal{V}_1$) nor in $\Xi_2$ (positively satisfied by $\mathcal{V}_2$), and so must be a negative formula in $\Xi_3$.

**Lemma 4.** $\Theta_3^-; \mathcal{V}^*(\Xi_3); \mathcal{V}^*(\Gamma_3^-) \vdash \mathcal{E}^*(\mathcal{A}_1|\mathcal{A}_2) : \mathcal{V}(\tau); \mathcal{V}^*(\Delta_3^-)$

*Proof.* Observe that $\mathcal{E}^* = (\mathcal{E}_1^* \cdot \mathcal{E}_1^*)^i$ and $\mathcal{V} = (\mathcal{V}_2 \cdot \mathcal{V}_1)^i$ for some $i \leq n$. Hence, it suffices to prove by induction on $i$ that $\Theta_2; (\mathcal{V}_2 \cdot \mathcal{V}_1)^i(\Xi_2); (\mathcal{V}_2 \cdot \mathcal{V}_1)^i(\Gamma_2^-) \vdash (\mathcal{E}_2^* \cdot \mathcal{E}_1^*)^i(\mathcal{A}_1|\mathcal{A}_2); (\mathcal{V}_2 \cdot \mathcal{V}_1)^i(\Delta_2^-)$.

Similarly, each term and continuation assigned to an output variable is well-typed under closure by $\mathcal{V}^*$ and $\mathcal{E}^*$ and thus:

**Proposition 5.** *$C_1|C_2 \fatsemi T_3$.*

It remains to show that the interaction conditions of Definition 5 are satisfied. The key is establishing condition 1 — that if $C_1 \xrightarrow{pl} C_1'$ and $C_2 \xrightarrow{\overline{pl}} C_2'$ then $T_1 \overset{pl}{\hookrightarrow} T_1'$ and $T_2 \overset{\overline{pl}}{\hookrightarrow} T_2'$ such that $T_1' \overset{T_2'}{\multimap} T_3$. This requires some further investigation of configuration types.

The interesting cases are those where $\mathcal{A}_1 \equiv \lambda x.t$ and $\mathcal{A}_2 \equiv K[\bullet s]$ (or vice-versa) and so they can perform the complementary actions $-\langle y, \alpha\rangle$ and $+\langle y, \alpha\rangle$. We need to show that $|\Theta_1|; |\Xi_1| \vdash \tau$ is *non-atomic* — that is, $|\Theta_1|; |\Xi_1| \vdash \tau = \forall X_1 \ldots X_m.\rho \to \sigma$ — for some $\rho, \sigma$. Observe that this implies that $|\Theta_2|; |\Xi_2| \vdash \tau$ is also non-atomic (since $\Xi_2$ contains the equations in $\Xi_1$) so that $T_1$ and $T_2$ can perform the complementary actions $-\langle y, \alpha\rangle$ and $+\langle y, \alpha\rangle$.

Since any derivation of a typing judgement for $\lambda x.t$ or $K[\bullet s]$ must conclude with $\to$-introduction followed by applications of the type-equality rule we have:

**Lemma 5.** *If $\Theta; \Xi; \Gamma \vdash \lambda x.t : \tau; \Delta$ or $\Theta; \Xi; \Gamma \vdash K[\bullet s] : \tau; \Delta$ then $\Theta; \Xi \vdash \tau$ is non-atomic.*

Hence, by the assumption that $(\mathcal{E}_1; \lambda x.t) \fatsemi (\Theta_1; \Xi_2; \Gamma_1 \vdash -\tau; \Delta_1)$ and $(\mathcal{E}_2; K[\bullet t]) \fatsemi (\Theta_2; \Xi_2; \Gamma_2 \vdash +\tau; \Delta)$ we know that $\Theta_1; \mathcal{V}_1(\Xi_1) \vdash \mathcal{V}_1(\tau)$ and $\Theta_2; \mathcal{V}_2^*(\Xi_2) \vdash \mathcal{V}_2^*(\tau)$ are non-atomic. From the latter we may infer that $\overline{\Theta_1}; \mathcal{V}_2^*(\overline{\Xi_1}) \vdash \mathcal{V}_2^*(\tau)$ is non-atomic, since $\Theta_2$ and $\Xi_2$ are interleavings of $\overline{\Theta_1}$ and $\overline{\Xi_1}$ with the disjoint contexts $\Theta_3$ and $\Xi_3$.

So to show that $|\Theta_1|; |\Xi_1| \vdash \tau$ is non-atomic is it is sufficient to prove the contrapositive.

**Lemma 6.** *Suppose $\mathcal{V}_+ \vDash_\Theta \Xi$ and $\mathcal{V}_- \vDash_{\overline{\Theta}} \overline{\Xi}$, where $|\Theta|; |\Xi| \vdash \tau$ is atomic. Then either $\Theta^-; \mathcal{V}_+(\Xi) \vdash \mathcal{V}_+(\tau)$ or $\Theta^+; \mathcal{V}_-(\Xi) \vdash \mathcal{V}_-(\tau)$ is atomic.*

*Proof.* We extend the grammar of types with an unbounded set of "neutral atoms" $A, B, C, \ldots$, which are equal only if syntactically identical, and prove the lemma for this extended set of types by an outer induction on the size of $\Theta$, and an inner induction on the sum of the lengths of the types in $\Xi$.

At least one of $\mathcal{V}_+(\tau)$ and $\mathcal{V}_-(\tau)$ must be atomic and so if $\Xi$ is empty then the hypothesis holds. Otherwise, $\Xi \equiv p(\sigma = \sigma'), \Xi'$ for some types $\sigma, \sigma'$ and equational context $\Xi'$ over $\Theta$, and polarity $p \in \{+, -\}$.

If $\sigma$ and $\sigma'$ are both non-atomic, then by satisfiability $\sigma \equiv \forall X_1 \ldots X_n.\rho_1 \to \rho_2$ and $\sigma \equiv \forall X_1 \ldots X_n.\rho_1' \to \rho_2'$ for some $\rho_1, \rho_2, \rho_1', \rho_2'$. Letting $A_1, \ldots, A_n$ be fresh, distinct atomic types, define $\widehat{\rho} = \rho[A_1/X_1, \ldots, A_n/X_n]$. The equational context $\Xi'' = p(\widehat{\rho_1} = \widehat{\rho_1}'), p(\widehat{\rho_2} = \widehat{\rho_2}'), \Xi'$ is equivalent to (satisfied by the same valuations as) $\Xi$, and so $\Theta; \Xi'' \vdash \tau$ is atomic, and positively and negatively satisfied by $\mathcal{V}_+$ and $\mathcal{V}_-$. Hence, by inner induction hypothesis, one of $\Theta^-; \mathcal{V}_+(\Xi'') \vdash \mathcal{V}_+(\tau)$ or $\Theta^+; \mathcal{V}_-(\Xi'') \vdash \mathcal{V}_-(\tau)$ is atomic.

Otherwise at least one of $\sigma$ and $\sigma'$ is atomic. If $\sigma \equiv \sigma'$, then we may discard the tautology $\sigma = \sigma'$ and apply the (inner) inductive hypothesis to $\Theta; \Xi' \vdash \tau$. Otherwise at least one of $\sigma, \sigma'$ must be a type-variable with polarity $p$ in $\Theta$ (none of the other cases are $p$-satisfiable). So assume without loss of generality that $\Theta \equiv \Theta', pX, \Theta''$ and $\Xi \equiv p(\sigma = X), \Xi'$. We may show that:

- $\Theta', \Theta''; \Xi'[\sigma/X] \vdash \tau[\sigma/X]$ is atomic.
- $\Theta, \Theta'' \vdash \Xi'[\sigma/X]$ is positively satisfied by $\mathcal{V}_+$ and negatively satisfied by $\mathcal{V}_-$.

So by the outer inductive hypothesis, either $(\Theta', \Theta'')^-; \mathcal{V}_+(\Xi[\sigma/X]) \vdash \mathcal{V}_+(\tau)$ or $(\Theta, \Theta'')^+; \mathcal{V}_-(\Xi[\sigma/X]) \vdash \mathcal{V}_-(\tau)$ is atomic, and hence either $\Theta^-; \mathcal{V}_+(\Xi) \vdash \mathcal{V}_+(\tau)$ or $\Theta^+; \mathcal{V}_-(\Xi) \vdash \mathcal{V}_-(\tau)$ is atomic.

We have shown that the arrow relation satisfies the first interaction condition. 2 and 3 are straightforward to verify, establishing that $(\mathsf{Comp}_{\lambda\mu2} \fatsemi \mathsf{Ty}_{\lambda\mu2})$ is a well-defined typed interaction structure. Therefore, by Proposition 1, typed bisimulation is preserved by parallel composition plus hiding, and thus:

**Theorem 1.** *Typed bisimulation is a congruence for the $\lambda\mu2$-calculus.*

## 5 Conclusions and Further Directions

We have described a "Curry-style" approach to game semantics, and used it to give new models of polymorphism. Various existing models may also be framed as typed interaction systems, such as the semantics of call-by-value in [12]. Nor are instances restricted to operational game semantics: for example we can present linear combinatory algebras of games and strategies in this way, and potentially other models of concurrent interaction. Unlike basic Church-style game semantics, these models give the opportunity to make finer distinctions between programs based on internal behaviour, which we have not explored here.

The notion of typed interaction structure reflects only limited structure of our models, but may be developed further. Having characterized parallel composition plus hiding within this setting, a natural next step would be a notion of copycat strategy, leading to structure for sharing and discarding information. One goal for such a development would be to put the generalization of congruence from configurations to terms on a systematic footing.

In another direction, our models of polymorphism may be developed further. In particular combining and fully exploiting generic and abstract data types often requires *higher-order* polymorphism, in which quantifiers range over *type operators* (functions which take types as arguments and return them as values). Whereas this is difficult to represent in game semantics, our model readily extends to a typing system based on System $F_\omega$, which allows quantification over type-operators: the price to pay is that satisfiability of configuration types (and thus effective presentation of the states of our LTS) requires the solution of higher-order unification problems, which are undecidable, in general.
